# Evaluation of the association of mucosa-associated invariant T (MAIT) cells with childhood asthma

**DOI:** 10.55730/1300-0144.5908

**Published:** 2024-07-07

**Authors:** Meral EKŞİ, Huri BULUT, Erdem AKALIN, Feyza USTABAŞ KAHRAMAN, Hakan YAZAN, Mebrure YAZICI, Mustafa Atilla NURSOY, Emin ÖZKAYA, Abdürrahim KOÇYİĞİT, Erkan ÇAKIR

**Affiliations:** 1 Department of Pediatrics, Faculty of Medicine, İstanbul University-Cerrahpaşa, İstanbul, Turkiye; 2 Department of Medical Biochemistry, Faculty of Medicine, Biruni University, İstanbul, Turkiye; 3 Department of Biology, Faculty of Medicine Hospital, Bezmiâlem Vakıf University, İstanbul, Turkiye; 4 Department of Pediatrics, Faculty of Medicine, Medipol University, İstanbul, Turkiye; 5 Division of Pediatric Pulmonology, Department of Pediatrics, Faculty of Medicine, Bezmiâlem Vakıf University, İstanbul, Turkiye; 6 Division of Pediatric Immunology and Allergy, Department of Pediatrics, Faculty of Medicine, Bezmiâlem Vakıf University, İstanbul, Turkiye; 7 Department of Medical Biochemistry, Faculty of Medicine, Bezmiâlem Vakıf University, İstanbul, Turkiye; 8 Division of Pediatric Pulmonology, Department of Pediatrics, Faculty of Medicine, İstinye University, İstanbul, Turkiye

**Keywords:** Mucosa-associated invariant T cells, childhood asthma, T helper 17 cell, innate cells, allergy, interleukin 17

## Abstract

**Background/aim:**

Innate-like T lymphocytes are a recently defined group of T cells comprising mainly mucosa-associated invariant T (MAIT) cells. The relationship between MAIT cells and childhood asthma is controversial. In this study, we aimed to determine the role of MAIT cells in patients with allergic asthma (AA) and nonallergic asthma (NAA). This is the first study to compare the ratios of these cells in patients with AA and NAA.

**Materials and methods:**

The study included children aged 6–18 years with AA (n = 41) or NAA (n = 30) and healthy control subjects (n = 36). The control group consisted of children who presented to the outpatient clinic without chronic disease, malnutrition, or acute or chronic infection. The proportions of MAIT, TH17, MAIT-17, and Th17-17 cells were investigated by flow cytometry and compared among the AA, NAA, and control groups.

**Results:**

When the AA and NAA patient groups were compared, the mean MAIT cell ratio was significantly lower in NAA patients (median: 0.45, p < 0.05). MAIT cell ratios were also substantially lower in NAA patients compared to the control group (mean: 0.504, p < 0.05). TH17, MAIT-17, and TH17-17 cell values were not statistically significant among the groups.

**Conclusion:**

Our study found that MAIT cell ratios were lower in the NAA patient group compared to the control group and AA patients. It has been predicted that MAIT cell depletion may have a role in the development of NAA. Our study is the first on this subject in the literature and further studies are needed.

## 1. Introduction

Asthma is a heterogeneous disease characterized by chronic airway inflammation associated with airway hyperresponsiveness to direct or indirect stimuli. It is defined by respiratory symptoms such as wheezing, shortness of breath, chest tightness and cough, and expiratory airflow limitation [1]. The most substantial risk factor identified in asthma development is the genetic predisposition of the immune system to develop specific IgE antibodies against atopy or common environmental allergens. While atopy has been described in the pathogenesis of asthma, other ways in which asthma develops without atopy are not clearly defined [2]. Many T-cell elements, cytokines, and immune mediators, especially eosinophils and mast cells, play roles in asthma development. Recent studies emphasize the heterogeneity of asthma pathogenesis. This heterogeneity may result from different pathogenic mechanisms, such as different patient phenotypes (symptoms, age of onset, atopy, and lung function), airway inflammation, remodeling, and immune and metabolic pathways in a particular microbial environment. Characteristic symptoms such as lung inflammation, mucus secretion, IgE production, and fibrosis, which occur mainly in cases of allergic asthma (AA), were found to be primarily associated with cytokines including interleukin (IL)-4, IL-5, and IL-13, produced by Th2 (T helper) cells [3].

In contrast, nonallergic asthma (NAA) is triggered by the neutrophil-dominant inflammatory response of past viral infections [4]. Neutrophil-dominant asthma is associated with IL-17 in mice and humans [5–7]. This cytokine has a role in initiating and maintaining neutrophilic inflammation [8]. It also contributes to airway smooth muscle remodeling with transforming growth factor beta (TGF-β) [9]. Recently, other potential pathways through which Th17 cells are involved in asthma’s pathogenesis have been suggested [10]. It has been reported that IL-17 expression in the circulation and airways increases with the severity of asthma in children and adults, and there is also higher IL-17 in the airways in patients with asthma exacerbations than in patients without asthma exacerbations [11–14]. While IL-17 expression was higher in blood and sputum supernatants obtained from children with AA and rhinitis compared to healthy controls, no significant difference was reported between children with severe asthma and the control group [15,16]. Although CD4+ T cells (Th17 cells) secreting IL-17 are thought to be the primary source of IL-17 in asthma, cellular sources such as invariant natural killer T (iNKT) and mucosal-associated invariant T (MAIT) cells also produce IL-17 [17,18]. Innate-like T lymphocytes (ILTs) are relatively recently identified T cells not involved in innate or adaptive immunity [19]. Humans have ILT lymphocyte populations with invariant T cell receptors and α-chains that recognize nonpeptide antigens, including iNKT and MAIT cells. There are few studies on the roles of iNKT and MAIT cells in asthma’s pathogenesis [3]. In recent studies, MAIT cells, a subset of ILT lymphocytes, were reported to have a role in the pathogenesis of asthma because of their presence in the lungs and their ability to produce Th2 cytokines [10]. However, little is known about the possible effects of MAIT cells on asthma’s pathophysiology in childhood.

Our aim in this study is to reveal the role of MAIT cells associated with the pathogenesis of asthma in the development of asthma in line with the most recent data by performing comparisons among AA, NAA, and control groups. Our study is the first to compare MAIT cell rates in these groups of patients.

## 2. Materials and methods

### 2.1. Patient and control groups

Seventy-one patients aged 6–18 years (31 girls) who applied to the Bezmiâlem Vakıf University Pediatric Chest Diseases Outpatient Clinic between January and December 2019 with three or more reactive airway attacks throughout their lives, typical asthma symptoms, and confirmed variable expiratory airflow limitations and were diagnosed with asthma according to the GINA 2018 criteria were included in this study. Thirty-six patients aged 6–18 years (23 girls) without any chronic diseases were included in the control group.

Patients with chronic upper airway cough syndrome, inhaled foreign body, bronchiectasis, primary ciliary dyskinesia, congenital heart disease, bronchopulmonary dysplasia, cystic fibrosis, immunodeficiency syndromes, neuromuscular disease, gastroesophageal reflux disease, or other causes of recurrent reactive airway attacks and those with other chronic diseases were excluded from the study.

The patient group was subdivided into patients with AA (n = 41) and NAA (n = 30) based on the GINA 2018 guidelines. Patients with at least one inhalant allergen sensitivity (dust, pollen, feathers, etc.) and personal and/or family histories of doctor-diagnosed eczema, allergic rhinitis, and food allergy were included in the AA group. In the NAA group, patients with no allergen sensitivity and no doctor-diagnosed allergic disease history and/or family history were included [2]. The control group consisted of healthy children presenting to the Pediatric Outpatient Clinic for routine control who did not have a history of reactive airway attack, malnutrition, or acute and/or chronic disease.

### 2.2. Flow cytometry

Peripheral venous blood samples from the AA, NAA, and control groups were collected in heparin tubes. Subsequently, 20 μL of CD3 FITC (Cat. No. 345763, BD, Franklin Lakes, NJ, USA), 20 μL of CD161 APC (Cat. No. 550968, BD), and 5 μL of TCR V alpha 7.2 PE (Cat. No. 351706, BioLegend, San Diego, CA, USA) antibodies were placed into 5-mL PS tubes of 12 × 75 mm and 100 μL of peripheral blood sample was added to each tube. After incubation in the dark at room temperature, FACS lysing solution (Ref. 349202, BD) was added to the tubes, and tubes were vortexed and incubated in the dark at room temperature for 5 min. After incubation, the tubes were centrifuged at 400 × *g* for 4 min and the supernatants were removed. Cell wash (Ref. 349524, BD) was added to the obtained pellets and vortexing was performed. All samples were centrifuged at 400 × *g* for 4 min and the supernatants were removed again, then vortexed with FACS permeabilizing solution (Ref. 340973, BD) and incubated in the dark at room temperature for 10 min. All supernatants were removed from the samples and subjected to recentrifugation and washing processes as described above. IL-17A PerCP 5.5 (Cat. No. 560799, BD) was then added to the tubes, which were incubated at room temperature in the dark for 3–40 min. All supernatants were removed after all samples were centrifuged and washed as described above, and then 200 μL of cell wash (Ref. 349524, BD) was added to the tubes and readings were done within 10 min. Analyses were performed with the BD FACSCanto II device using BD FACSDiva software.

### 2.3. Statistical analysis

For statistical analysis, IBM SPSS Statistics 22.0 (IBM Corp., Armonk, NY, USA) was used. The distribution of the data was analyzed using the Shapiro–Wilk test. The Mann–Whitney U test was used to compare two groups without normal distribution. The Kruskal–Wallis test was used in comparisons of three groups without normal distribution. Post hoc comparisons of significant results were performed with Dunn’s test. Descriptive statistics were given as median, interquartile range, and mean ± standard deviation values. Significance was accepted at p < 0.05.

Ethics committee approval for this study was obtained from the Bezmiâlem Vakıf University Clinical Research Ethics Committee.

## 3. Results

### 3.1. Patient and control groups

The mean age of the 41 AA patients (48.7% female) was 10.5 ± 3.1 years, while the mean age of the 30 patients (40% female) diagnosed with NAA was 8.2 ± 2.3 years. All patients in the AA group had positive allergy skin tests, while the allergy skin tests of all patients in the NAA group were negative. In the control group, 63.9% of the children were girls and the mean age was 10.9 ± 3.2 years. There were no significant differences between any groups in terms of mean age or sex. The demographic and clinical data of all patients and control subjects are summarized in Table 1.

### 3.2. Flow cytometry results and comparisons

When the patient and control groups were compared, MAIT cell ratios were significantly lower in NAA patients (p = 0.011) (Table 2; Figure). When the means of Th17, MAIT+ IL-17A+, and CD3+ IL-17A+ cell ratios were evaluated, however, no significant differences were found among the groups (p = 0.319, p = 0.568, and p = 0.467) (Table 2). When the NAA and control groups were compared, the mean MAIT cell ratio was significantly lower in the NAA group (p = 0.011). No significant difference was found when the mean Th17, MAIT+ IL-17A+, and CD3+ IL-17A+ cell ratios were compared between the NAA and control groups (p > 0.05). When the AA and control groups were compared, significant differences were not found between the groups for Th17, MAIT, MAIT+ IL-17A+, or CD3+ IL-17A+ cell ratios (p > 0.05) (Table 2). Finally, when comparing the mean Th17, MAIT, MAIT+ IL-17A+, and CD3+ IL-17A+ cell ratios of all patients (AA + NAA) and the control group, no significant differences were found (p > 0.05).

## 4. Discussion

Although asthma is a prevalent disease in children, there is little information about the role of MAIT cells in the pathophysiology of childhood asthma. Our study compared MAIT cells, Th17 cells, and the IL-17 levels produced by them in AA and NAA patients and a control group in the pediatric population.

In the NAA group, MAIT cell levels were significantly lower than in the other groups. When we compared the mean ratios of IL-17-producing MAIT cells (MAIT+ IL-17A+) between the groups, we did not find a significant difference.

MAIT cells were recently identified and their associations with bacterial, fungal, and mycobacterial infections and autoimmune diseases have been demonstrated [20]. Asthma pathogenesis is very heterogeneous, and many cytokines and cell groups have been associated with asthma in recent years. It has been shown in the literature that iNKT and MAIT cells are associated with asthma [21]. One study suggested that high levels of circulating MAIT cells at 1 year of age reduce the risk of developing asthma at 7 years. High circulating MAIT cells are highly correlated with IFN-γ-producing CD4+ T cells and were suggested to be protective against asthma development [22]. In the study by Lezmi et al., school-aged asthmatic patients with and without attacks were evaluated and it was found that MAIT-17 cells had a positive correlation with asthma severity. The authors attributed this to MAIT-17 causing asthma symptoms [10]. In a study conducted by the same team with children with severe asthma in 2019, MAIT-17 and TH17 were evaluated in bronchoalveolar lavage (BAL) fluid and it was shown that the amount of those cells in BAL increased more than in the blood [23]. In our study, MAIT cells were low in the NAA group, but the relationship between disease severity and MAIT cells was not evaluated.

Likewise, regardless of the patient’s asthma severity, there was no significant difference between the MAIT-17 and TH17 values in the AA, NAA, and control groups. IL-17 levels were previously evaluated in the nasal/bronchial mucosa of adult patients with severe and mild asthma and it was shown that IL-17 expression was higher in cases of severe asthma. Furthermore, it was established in that study that IL-17 was associated with bronchial neutrophilia, attacks, and forced expiratory volume in the first second [24]. In our study, plasma was sampled instead of the nasal mucosa and we found no significant difference in numbers of IL-17-producing MAIT cells in the AA, NAA, and control groups. A study by Ashmore et al. in adults showed that MAIT cells were negatively correlated with airway limitations in patients with asthma [25].

Our study had some limitations. In particular, the patients’ clinical grades, attack statuses, and asthma scores were not evaluated. In future studies, it would be beneficial to investigate MAIT cells at the tissue level (i.e., sputum or BAL fluid) and during acute attacks. Detailed analyses of the frequency and functional subgroups of these cells in the context of different asthma endotypes could be crucial for the development of new therapeutic approaches.

In conclusion, this study found that MAIT cell values were lower in patients with NAA compared to patients with AA and the control group. This is the first study in the literature to compare MAIT cells in children with AA and NAA.

## Figures and Tables

**Figure:**
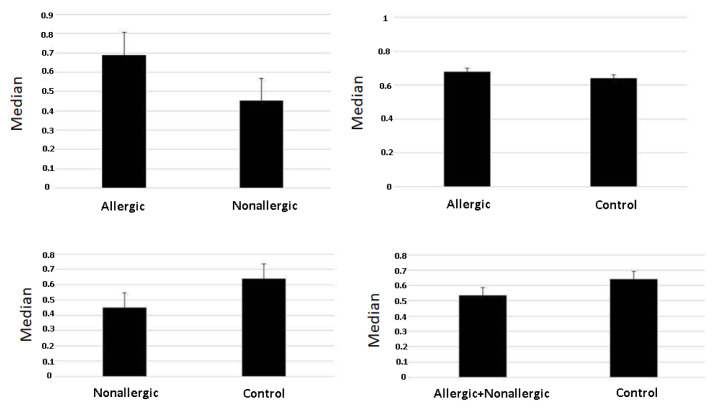
Comparison of MAIT results of the allergic asthma, nonallergic asthma, and control groups.

**Table 1:** Demographic and clinical characteristics of the patient and control groups.

	AA (n: 41)	NAA (n : 30)	Control (n : 36)	p
**Age, years**	10.5 ± 3.1	8.2 ± 2.3	10.9 ± 3.2	> 0.05*
**Girls (n, %)**	20, 48.7%	12, 40%	23, 63.9 %	>0.05*
**Typical asthma symptoms (n, %)**	41, 100%	30, 100%	0	>0.05*
**Confirmed variable expiratory airflow limitation (n,%)**	41, 100%	30, 100%	0	>0.05*
**Skin prick test positivity (n, %)**	41, 100%	0	0	>0.05*
**Family history of atopy**	35, 87.5%	0	0	
**Total IgE height (n, %)**	36, 90%	0	0	
**Eosinophilia (n, %)**	25, 62.5%	0	0	

* Pearson chi-square test,

AA: allergic asthma, NAA: nonallergic asthma.

**Table 2:** Comparison of the results of the allergic asthma, nonallergic asthma, and control groups.

	MAIT*	MAIT + IL 17 A +*	TH17*	CD3+IL17A+*
**Allergic asthma (**mean):	0.80 ± 0.52	0.018 ± 0.012	22.68 ± 6.22	0.19 ± 0.14
**Allergic asthma Median** (IQR):	0.68 (0.06–2.42)	0.02 (0.005–0.05)		0.15 (0.03–0.74)
**Nonallergic asthma** (mean)	0.50 ± 0.33	0.016 ± 0.011	22.16 ± 6.73	0.14 ± 0.07
Median (IQR)	0.45 (0.05–1.4)	0.015 (0–0.04)		0.13 (0.02–0.34)
**Control** (mean):	0.82 ± 1.00	0.024 ± 0.020	23.96 ± 8.39	0.25 ± 0.42
Median (IQR)	0.64 (0.22–6.3)	0.02 (0–0.09)		0.15 (0.02–2.46)
p* **value**	0.027	0.319	0.568	0.467
**Post hoc** p^**^ **value**	p^a^,^n^ = 0.011	p^a^,^n^ = 0.66	p^a^,^n^ = 0.736	p^a^,^n^ = 0.99
	p^a^,^c^ = 0.526	p^a^,^c^ = 0.264	p^a^,^c^ = 0.451	p^a^,^c^ = 0.787
	p^n^,^c^ = 0.039	p^n^,^c^ = 0.145	p^n^,^c^ = 0.594	p^n^,^c^ = 0.779

MAIT: Mucosa-associated invariant T cell, MAIT + IL17A+: Interleukin 17 A producing MAIT, TH17: T helper 17, CD3+IL17A+: Interleukin 17 A producing CD 3 lymphocyte,

* Kruskal–Wallis test,

a atopic asthma,

n nonatopic asthma,

c control.
